# Effect of phosphorus deficiency on photosynthetic inorganic carbon assimilation of three climber plant species

**DOI:** 10.1186/s40529-014-0060-8

**Published:** 2014-08-01

**Authors:** Deke Xing, Yanyou Wu

**Affiliations:** 1 Research center for Environmental Bio-Science and Technology, State Key Laboratory of Environmental Geochemistry, Institute of Geochemistry, Chinese Academy of Sciences, Guiyang, 550002 China; 2 Graduate School of Chinese Academy of Sciences, Beijing, 100049 China

**Keywords:** Adaptation mechanism, Carbonic anhydrase, Chlorophyll fluorescence, Composition of the stable carbon isotope, Photosynthesis

## Abstract

**Background:**

P deficiency in karst areas significantly influenced leaf photosynthesis and carbon metabolisms in plants which were bad for plant growth. Meanwhile, fertilizer application would cause lots of environmental problems. Therefore planning and developing P deficiency-resistant plants in karst areas are important to prevent shortage of P resources and reduce the environmental impacts of P supplementation.

**Results:**

This study examined the photosynthetic response of three climber plant species, namely, *Pharbitis nil* (Linn*.*) Choisy, *Lonicera pampaninii* Levl, and *Parthenocissus tricuspidata* (Sieb.et Zucc*.*) Planch to phosphorus (P) deficiency stress. The plants were exposed to P deficiency stress at three treatments of 0.125 mM, 0.031 mM, and 0 mM for 30 d; 0.250 mM P was used as the control. Photosynthetic responses were determined by measurement of leaf photosynthesis, chlorophyll fluorescence, carbonic anhydrase activity, and stable carbon isotope ratios. *Pharbitis nil* showed high CA activity, more negative δ^13^C values and could maintain long-term stable photosynthetic capacity. *Lonicera pampaninii also showed high CA activity but positive* δ^13^C values compared to *Pharbitis nil, and its* photosynthetic capacity decreased as P deficiency stress increased. *Parthenocissus tricuspidata* had a low photosynthesis and positive δ^13^C values compared to *Pharbitis nil,* it could grow normally even under 0 mM P.

**Conclusions:**

*Pharbitis nil* was tolerant to long-term, severe P deficiency stress, a finding that is attributed to its stable PSII and regulation of carbonic anhydrase. *Lonicera pampaninii* showed a poor adaptability to short-term P deficiency, but exhibited long-term tolerance under 0.125 mM P concentration. *Parthenocissus tricuspidata* was tolerant to long-term P deficiency stress, may exhibit a stomatal limitation. Besides, P deficiency stress had little effect on the way of inorganic carbon utilization of the three climber plants. Different adaptation mechanisms to P deficiency stress should be considered for the selection of species when developing P deficiency-resistant plants.

**Electronic supplementary material:**

The online version of this article (doi:10.1186/s40529-014-0060-8) contains supplementary material, which is available to authorized users.

## Background

Phosphorus (P) is an essential macronutrient for plant growth and development. It is a component of several cellular molecules, such as ATP, nucleic acids, phospholipids, and phosphorylated sugars, and thus plays a crucial role in carbon metabolism (Huang et al., [2008]). However, inorganic P is one of the least available nutrients in the soils of several terrestrial ecosystems (Vance et al., [2003]), which occasionally leads to P deficiency, especially in karst areas. P deficiency in modern agricultural systems can be alleviated by fertilizer application, but fertilizer costs are variable and concerns have been raised about their potential environmental impacts (Zhang et al., [2009]). The continuing demand for P could deplete global P reserves by the end of the century (Byrne et al., [2011]). Thus, improvements in P acquisition and P use efficiency are becoming increasingly important to prevent shortage of P resources and reduce the environmental impacts of P supplementation.

P deficiency induces a wide array of metabolic effects that limit plant growth. Hogh-Jensen et al. have reported that a low P status induces changes in the relative growth of the roots and shoots rather than changes in the carbon uptake rates per unit mass or area of these organs (Hogh-Jensen et al., [2002]). Usuda and Shimogawara ([1995]) showed that the soluble and insoluble protein contents of phosphorus-deficient maize decreased compared with that of the control plants. Other researchers reported that P deficiency significantly influences leaf photosynthesis and carbon metabolisms in plants (Rao, [1996]; Foyer and Spencer, [1986]; Fredeen et al., [1989]; Rao and Terry, [1995]). Inhibition of photosynthesis caused by P deficiency is mainly due to the decrease in the ribulose-1,5-bisphosphate (RuBP) pool size (Jacob and Lawlor, [1992]; Pieters et al., [2001]). But under stress conditions, carbonic anhydrase (CA) could always provide carbon and water source for the photosynthesis process, and CA is involved in diverse physiological processes, such as ion exchange, acid–base balance, CO_2_ transfer, respiration, biosynthesis, and photosynthetic CO_2_ fixation (Badger and Price, [1994]; Sasaki et al., [1998]). Increased CA activity of *Chlorella vulgaris* under P deficiency facilitated the cellular mechanism of dissolved inorganic carbon (DIC) concentration and enhanced the CO_2_ influx to the site of Rubisco (Kozlowska-Szerenos et al., [2000]).

Chlorophyll *a* fluorescence (ChlF) may assess the integrity and efficiency of the photosynthetic apparatus and the overall health of the plant tissue (Roháček and Barták, [1999]). Changes in ChlF emissions, arising mainly from PSII, provide information on almost all aspects of photosynthetic activity. Therefore, ChlF had also widely been used to probe photosynthetic function in higher plants and exhibit plant tolerance to environmental stresses (Gray et al., [2006]; Guo et al., [2005]; Panda et al., [2008]).

The stable isotope technique is an important tool to identify the source of an element. The ratios of stable carbon isotopes δ^13^C have been successfully used to study photosynthesis (Motomura et al., [2008]; Schwender et al., [2004]; Tcherkez et al., [2009]). The ratios of stable carbon isotopes δ^13^C in plants change when the carbon metabolic pathways and the sources of inorganic carbon consumed for photosynthesis are changed. The labeling of the stable carbon isotopes in exogenous bicarbonate can trace whether plants obtain CO_2_ from the conversion of bicarbonate through the action of CA or not. Use of bicarbonate labeled with stable carbon isotopes and the determination of stable isotopes may yield the bicarbonate utilization proportion in *Broussonetia papyrifera* (L.) Vent. under treatment with high concentrations (10 mM) of bicarbonate (Wu and Xing, [2012]). Therefore, δ^13^C were measured to trace the metabolic route of the inorganic carbon source during photosynthesis (Motomura et al., [2008]).

The Japanese morning glory (*Ipomoea nil* (L.) Roth. or *Pharbitis nil* (L.) Choisy) was first introduced to Japan from China over 1000 years ago as a medicinal herb (Kajita and Nishino, [2009]). *P. nil* is a short-day plant requiring a single long dark period for floral induction. It is used as a model plant for photoperiodic flower induction studies (Nishino, [1976]; Reese and Erwin, [1997]).

Dried buds of several species of the genus *Lonicera* (*Caprifoliaceae*) are commonly used in traditional Chinese medicine for latent-heat-clearing, antipyretic, detoxicant, and anti-inflammatory properties. Several reports have shown that *Flos Lonicerae* possess effective antioxidant properties (Ku et al., [2009]; Xiang and Ning, [2008]). *Parthenocissus tricuspidata* (Sieb. et Zucc.) Planch is a vertical virescent medicinal plant of the Vitaceae family that can climb to heights of 20 m or higher through attachment of its adhesive tendrils to supports (Kim et al., [2005]; Wang et al., [2010]). Several reports about these three climber plant species are available, *Lonicera pampaninii* is a pioneer species in the karst mountain areas where the vegetations were generally at P-limited stress (Du et al., [2011]), *P. nil* and *P. tricuspidata* can also grow in the karst areas, however, distribution of the three climber plant species are different, so it is necessary to study their photosynthetic responses to P deficiency stress or their photosynthetic adaptation mechanisms in response to P deficiency stress.

Therefore, the aim of this study was to understand the photosynthetic inorganic carbon assimilation capacity of these three C3 plant species under P deficiency stress, and photosynthetic characteristics, ChlF parameters, CA activity under P deficiency stress were determined. Besides, the utilization of inorganic carbon sources was studied by comparing the differences between these three climber plants in terms of the foliar composition of the stable carbon isotope. The different adaptation mechanisms discovered could provide a general consideration for the planning and development of P deficiency-resistant plants.

## Methods

### Plant growth and P deficiency stress treatment

The experiment was conducted in a growth chamber at the Institute of Geochemistry, Chinese Academy of Sciences, Guizhou Province, China (26.35°N, 106.42°E). Seedlings of *Pharbitis nil* (Linn*.*) Choisy, *Lonicera pampaninii* Levl, and *Parthenocissus tricuspidata* (Sieb.et Zucc*.*) Planch were germinated and cultivated in 12-hole trays with quartz sand under a 12 h photoperiod (200 μmol m^−2^ s^−1^ PPFD), a day/night temperature cycle of 28°C /20°C, and 60% relative humidity. Plants were irrigated daily with 1/4-strength Hoagland solution (Hoagland and Arnon, [1950]). After 75 d of growth, the nutrient solution was replaced by a modified Hoagland solution containing 6 mM KNO_3_, 4 mM Ca(NO_3_)_2_, 2 mM MgSO_4_, 2 mM Fe(Na)EDTA, 2 μM KCl, 50 μM H_3_BO_3_, 4 μM MnSO_4_, 4 μM ZnSO_4_, 0.2 μM CuSO_4_, and 0.2 μM (NH_4_)_6_MO_7_O_24_ at pH 8.1 ± 0.5. The solution was supplemented with 10 mM NaHCO_3_ which δ^13^C was −17.23‰ Pee Dee Belemnite. Three P deficiency stress treatments of 0.125 mM, 0.031 mM, and 0 mM were simulated by varying concentration combinations of NH_4_H_2_PO_4_ and NH_4_Cl; 0.250 mM P was used as the control. Seedlings that germinated healthily and uniformly were subjected to simulated P deficiency stress. The experiment was arranged in a completely randomized design, and 24 healthy and uniform seedlings from each species of climber plant were used for each stress condition. P deficiency treatment lasted for 30 d, during which the solution was changed every other day. Measurements were done in triplicate on days 10, 20, and 30.

### Net CO_2_ assimilation rate measurements

Net CO_2_ assimilation rate (An) were measured on the fourth youngest fully expanded leaf from the top at 9:30 a.m. to 11:00 a.m. every 10 d from the onset of P deficiency. Three plants from each treatment group were subjected to measurement using a portable LI-6400XT photosynthesis measurement system (LI-COR, Lincoln, NE, USA). The photosynthetic active radiation (PAR), temperature, and CO_2_ concentration during the measurements were 300 μmol m^−2^ s^−1^, 30°C, and 400 μmol mol^−1^, respectively.

### Fluorescence measurements

Chlorophyll *a* was measured using a portable LI-6400XT photosynthesis measurement system. Leaves were adapted to the dark for 30 min to ensure complete relaxation of all reaction centers before measurement. The fourth youngest fully expanded leaf from the top was selected for the measurement every 10 d after the onset of P deficiency. Three plants from each treatment group were used for the measurement. The minimum ChlF (F_0_) was determined using a measuring beam, whereas the maximum ChlF (Fm) was recorded after exposure to a 0.8 s saturating light pulse (6000 μmol m^−2^ s^−1^). Then plant leaves were light-induced by 1000 μmol m^−2^ s^−1^ radiation intensity light for 30 min. Actinic light (300 μmol m^−2^ s^−1^) was then applied for 1 min to drive photosynthesis. The maximum fluorescence in the light-saturated stage (F′m), basic fluorescence after induction (F′_0_), and fluorescence yield in the steady state (Fs) were determined. The maximum quantum yield of PSII (Fv/Fm) was calculated as (Fm – F_0_)/Fm, and the effective quantum yield of PSII (Φ_PSII_) was calculated as ΔF/F′m = (F′m – Fs)/F′m).

### Carbonic anhydrase activity

The fourth and fifth youngest fully expanded leaves from the top were chosen for CA activity measurement every 10 d after the onset of P deficiency. Three plants from each treatment group were used for the measurement. Leaf tissues (0.1 g to 0.2 g) were quickly frozen in liquid nitrogen and ground with 3 mL extraction buffer (0.01 M barbitone sodium with 0.05 M mercaptoethanol, pH 8.3). The homogenate was centrifuged at 10000 × g and 0°C for 5 min and then placed on ice for 20 min. The supernatant was used to determine CA activity using the pH method described by Wilbur and Anderson ([1948]) with modifications (Wu et al., [2011]). In brief, CA activity was assayed at 0°C to 2°C in a mixture containing 4.5 mL of 0.02 M barbitone buffer (5, 5-diethylbarbituric acid; pH 8.3), 0.4 mL of the sample, and 3 mL of CO_2_-saturated H_2_O. CA activity was expressed in Wilbur and Anderson (WA) units as WA = (t_0_/t)–1, where t_0_ and t are the time(s) measured for the pH change (8.2 to 7.2), with buffer alone (t_0_) and with sample (t).

### Stable carbon isotope ratios measurements

The stable carbon isotope ratio (δ^13^C) was determined from the first youngest fully expanded leaf from the top using gas isotope ratio mass spectrometry (Mat-252, Finnigan MAT, Germany). Four expanded leaves at each stress treatment in each species of climber plant were randomly detached from the 24 seedlings every 10 d after the onset of P deficiency.

### Statistical analysis

All measurements were subjected to analysis of variance (ANOVA) to discriminate significant differences (defined as P ≤ 0.05) between group means. Data are shown as the mean ± standard error (SE) (n = 5). These mean data were analyzed statistically using a factorial design through SPSS software (version 13.0, SPSS Inc.), and mean results were compared through LSD post hoc test at 5% significance level (p < 0.05).

## Results

### Net CO_2_ assimilation rate

**Figure 1:**
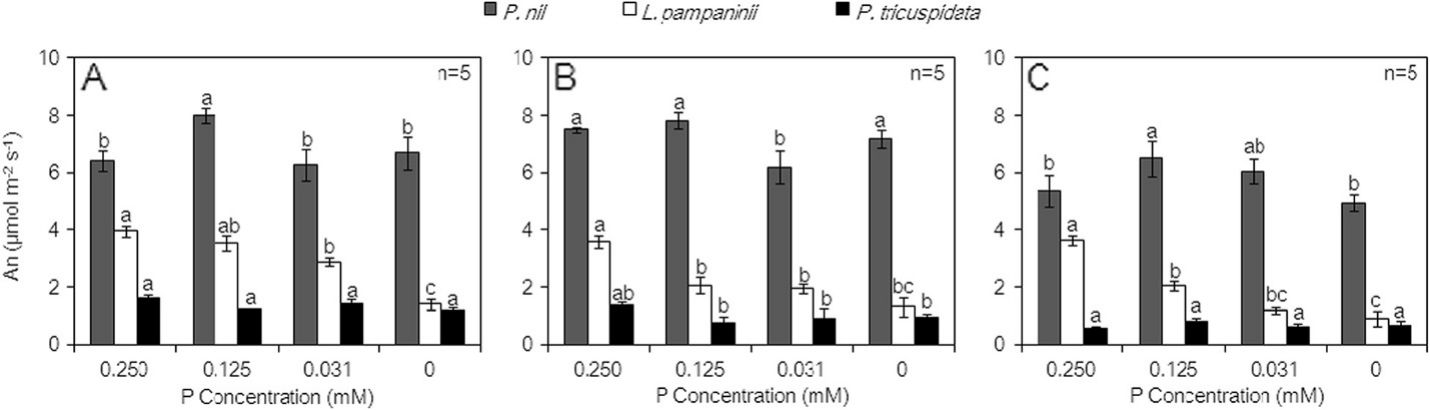
**Effects of P deficiency stress on the An of the three climber plant species (A. day 10, B. day 20, C. day 30).** Mean ± SE (n = 5) followed by different letters in the same species and in the same treatment period indicate significant difference at P ≤ 0.05, according to one-way ANOVA and t-test.

Figure 1 shows the An of the three climber species; An varied with plant species, P deficiency stress, and stress duration. The An of *P. nil* was higher than those of the two other species. *P. tricuspidata* showed the lowest An, and no significant change of An with decreasing P concentration and increasing stress duration for *P. tricuspidata* was observed over the entire duration of P deficiency stress. On day 10, the An of *P. nil* under the 0.125 mM P treatment was higher than those in the other treatments, which showed no significant change. The An of *L. pampaninii* under the 0 mM P treatment was 35.62% that under the control; its An under the 0.031 mM P treatment was also lower than those under the control and 0.125 mM P treatments (Figure 1A). On day 20, the An of *P. nil* under the 0.031 mM P treatment was lower than those under other treatments. The An of *L. pampaninii* under the control treatment was higher than those in other treatments, which showed no significant change (Figure 1B). On day 30, the An of *P. nil* under the 0.125 mM P treatment was higher than those under other treatments, which showed no significant change. The An of *L. pampaninii* under the 0 mM P treatment was 24.36% of the value under the control; its An under the 0.031 mM P treatment was also lower than those under the control and 0.125 mM P treatments (Figure 1C).

### Chlorophyll fluorescence

Table 1 shows the maximal PSII photochemical efficiency (Fv/Fm) of the three species. The Fv/Fm values of *P. nil* and *P. tricuspidata* were not changed markedly with increasing P deficiency stress over the entire P deficiency stress duration, whereas the Fv/Fm of *L. pampaninii* showed a lower value under the 0 mM P treatment.

**Table 1:** **Effects of P deficiency stress on the Fv/Fm of the three climber plant species**

Stage	Material	P concentration (mM)			
		0.250	0.125	0.031	0
10th day	*P. nil*	0.77 ± 0.016ab	0.80 ± 0.006a	0.79 ± 0.007a	0.80 ± 0.001a
	*L. pampaninii*	0.79 ± 0.002a	0.79 ± 0.010a	0.77 ± 0.011a	0.74 ± 0.027b
	*P. tricuspidata*	0.79 ± 0.005a	0.78 ± 0.012a	0.76 ± 0.006ab	0.78 ± 0.005a
20th day	*P. nil*	0.80 ± 0.008a	0.80 ± 0.002a	0.77 ± 0.028ab	0.80 ± 0.002a
	*L. pampaninii*	0.78 ± 0.011a	0.78 ± 0.001a	0.77 ± 0.004ab	0.73 ± 0.038b
	*P. tricuspidata*	0.78 ± 0.009a	0.77 ± 0.014ab	0.78 ± 0.002a	0.77 ± 0.014ab
30th day	*P. nil*	0.75 ± 0.034a	0.75 ± 0.013a	0.75 ± 0.018a	0.78 ± 0.009a
	*L. pampaninii*	0.79 ± 0.005a	0.79 ± 0.002a	0.78 ± 0.005a	0.74 ± 0.010b
	*P. tricuspidata*	0.76 ± 0.002a	0.77 ± 0.009a	0.78 ± 0.002a	0.78 ± 0.002a

Mean ± SE (n = 5) followed by different letters in the same species and in the same treatment period indicate significant difference at P ≤ 0.05, according to one-way ANOVA and t-test.

Table 2 shows the effective quantum yield of PSII (Φ_PSII_) of the two species. On days 10 and 20, the Φ_PSII_ of *P. nil* and *P. tricuspidata* did not change markedly with increasing P deficiency stress; however, the Φ_PSII_ of *L. pampaninii* showed a low value under the 0 mM P treatment. On day 30, the Φ_PSII_ of *P. nil* showed a high value under the 0 mM P treatment, the Φ_PSII_ of *P. tricuspidata* did not change markedly with increasing P deficiency stress, and the Φ_PSII_ of *L. pampaninii* showed a low value under the 0 mM P treatment.

**Table 2:** **Effects of P deficiency stress on the** Φ_**psII**_**of the three climber plant species**

Stage	Material	P concentration (mM)			
		0.250	0.125	0.031	0
10th day	*P. nil*	0.25 ± 0.019ab	0.26 ± 0.046a	0.21 ± 0.023b	0.24 ± 0.052b
	*L. pampaninii*	0.27 ± 0.014ab	0.34 ± 0.013a	0.24 ± 0.034bc	0.16 ± 0.048c
	*P. tricuspidata*	0.15 ± 0.004a	0.14 ± 0.017a	0.15 ± 0.011a	0.15 ± 0.012a
20th day	*P. nil*	0.28 ± 0.004a	0.25 ± 0.015ab	0.25 ± 0.018ab	0.24 ± 0.054ab
	*L. pampaninii*	0.22 ± 0.020a	0.25 ± 0.011a	0.20 ± 0.012ab	0.11 ± 0.045c
	*P. tricuspidata*	0.14 ± 0.008a	0.11 ± 0.025ab	0.15 ± 0.003a	0.16 ± 0.007a
30th day	*P. nil*	0.26 ± 0.015b	0.23 ± 0.040b	0.26 ± 0.039ab	0.34 ± 0.015a
	*L. pampaninii*	0.22 ± 0.020ab	0.23 ± 0.046a	0.22 ± 0.035ab	0.16 ± 0.030b
	*P. tricuspidata*	0.13 ± 0.007a	0.14 ± 0.009a	0.12 ± 0.007a	0.15 ± 0.019a

Mean ± SE (n = 5) followed by different letters in the same species and in the same treatment period indicate significant difference at P ≤ 0.05, according to one-way ANOVA and t-test.

### Carbonic anhydrase activity

**Figure 2:**
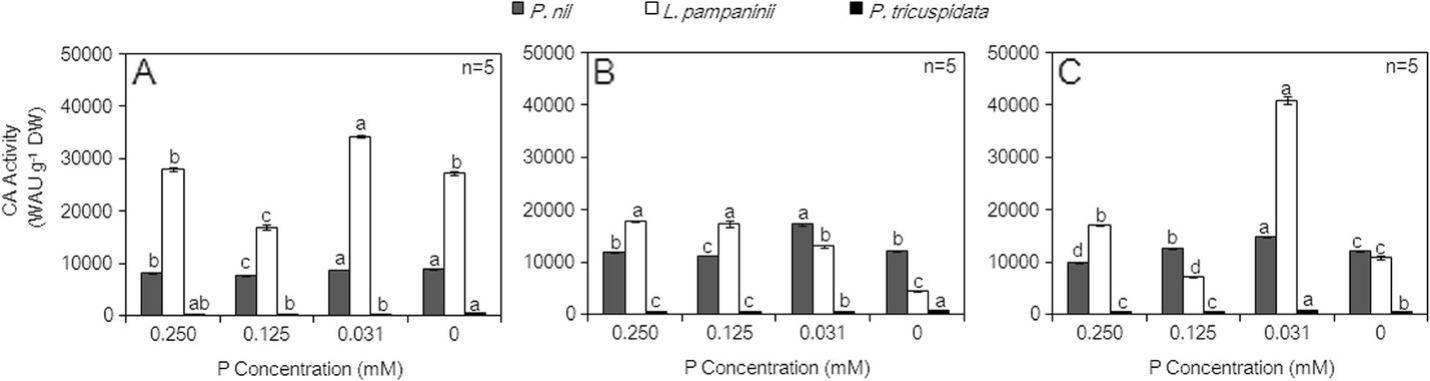
**Effects of P deficiency stress on the CA activities of the three climber plant species (A. day 10, B. day 20, C. day 30).** Mean ± SE (n = 5) followed by different letters in the same species and in the same treatment period indicate significant difference at P ≤ 0.05, according to one-way ANOVA and t-test.

CA activity varied with plant species, P deficiency stress level, and durations. CA activity was higher in *P. nil* and *L. pampaninii* than in *P. tricuspidata*, for which CA activity could hardly be determined and remained consistently low (Figure 2). On day 10, among the treatments, *L. pampaninii* showed the highest CA activity under 0.031 mM P treatment and the lowest value under 0.125 mM P treatment, which was 49.25% of the value under the 0.031 mM P treatment. The CA activity of *P. nil* under 0.031 or 0 mM P treatment was higher than those under other treatments. Moreover, the CA activity of *P. nil* was always lower than that of *L. pampaninii* at all treatments (Figure 2A). On day 20, the CA activity of *P. nil* under the 0.031 mM P treatment was higher than those under other treatments. The CA activity of *L. pampaninii* under 0.125 mM P treatment or control was higher than those under other treatments. The CA activity of *L. pampaninii* under the 0 mM P treatment showed the lowest value at only 25.34% of the value under the 0.125 mM P treatment (Figure 2B). On day 30, the CA activity of *P. nil* under the 0.125 and 0.031 mM P treatments was higher than those under the 0 mM P concentration or control treatments; the value under the 0.031 mM P treatment was the highest. *L. pampaninii* also showed the highest CA activity under the 0.031 mM P treatment; this value was the highest among all the species studied. The CA activity of *L. pampaninii* under the 0.125 mM P treatment was lower than those under other treatments (Figure 2C). In addition, the CA activity of *L. pampaninii* under the 0 mM P treatment on day 30 was higher than that on day 20.

### Carbon stable isotope ratios

**Figure 3:**
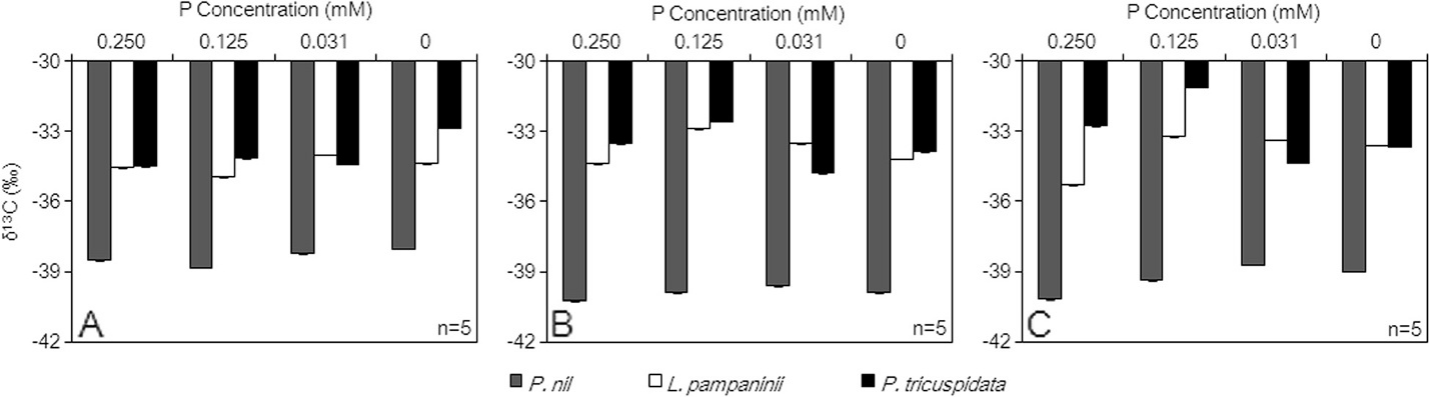
**Effects of P deficiency stress on the δ** ^**13**^ **C values of the three climber plant species (A. day 10, B. day 20, C. day 30).**

The δ^13^C value was significantly lower in *P. nil* than in the two other species. The δ^13^C values for *P. nil* were all lower than −38‰, whereas those of *L. pampaninii* and *P. tricuspidata* were around −34‰ and higher. On day 10, the δ^13^C values of *P. nil* and *L. pampaninii* under the 0.031 mM and 0 mM P treatments were a little higher than those under control or 0.125 mM P treatments. The δ^13^C value of *P. tricuspidata* under the 0 mM P treatment was more positive than those under the control, 0.125 mM, and 0.031 mM P treatments (Figure 3A). On day 20, the δ^13^C values of *P. nil* showed no significant change with increasing P deficiency stress, whereas those of *L. pampaninii* and *P. tricuspidata* showed more positive δ^13^C values under the 0.125 mM P treatment. In addition, the δ^13^C value of *P. tricuspidata* under the 0.031 mM P treatment was more negative than those of *P. tricuspidata* under the three treatments (Figure 3B). On day 30, the δ^13^C values of *P. nil* under the 0.031 mM and 0 mM P treatments were slightly higher than those under control or 0.125 mM P treatments. The δ^13^C value of *L. pampaninii* under the control treatment was more negative than those under the 0.125 mM, 0.031 mM, and 0 mM P treatments. *P. tricuspidata* showed the highest δ^13^C value under the 0.125 mM P treatment, and its δ^13^C values under the 0.031 mM and 0 mM P treatments were slightly lower than those under the control or 0.125 mM P treatments (Figure 3C).

## Discussion

### *Pharbitis nil*(Linn*.*) Choisy

The photosynthetic rate of plants could be affected by a non-stomatal factor that primarily depends on the activity of intrinsic enzymes, photosynthetic apparatus, and their regulation mechanisms (Li et al., [2006]). P deficiency could result in smaller in size of stomatal opening (Sarker et al., [2010]), atmospheric CO_2_ became hard to entry into cell of plant, but through catalysis of CA in *P. nil*, which showed high activity, another carbon source could be supplied for photosynthesis of *P. nil* by the transformation from HCO_3_^−^ to CO_2_ under P deficiency stress. Photosynthesis efficiency can be described by the maximal PSII photochemical efficiency (Fv/Fm) and actual photochemical quantum efficiency of open PSII (Φ_PSII_). The response of Fv/Fm and Φ_PSII_ in *P. nil* to increased P deficiency indicated that *P. nil* was tolerant to 0 mM P concentration. *P. nil* maintained higher quantum efficiencies in the primary reaction center of the open PSII with long-term P deficiency stress durations, especially under 0 mM P. In other words, *P. nil* showed long-term tolerance to P deficiency stress. *P. nil* could maintain long-term high and stable photosynthetic inorganic carbon assimilation ability even under 0 mM P concentration.

Photosynthesis of plants is the most important process in carbon isotopic fractionation in the nature, the more negative the δ^13^C value in carbon source of plants photosynthesis and the higher the photosynthetic rate were, the more negative the δ^13^C value in plants leaves was. In fact, after dissolved in the nutrient solution, δ^13^C value of atmospheric CO_2_ was about −11‰ (Clark and Fritz [1997]), δ^13^C values of naturally grown C3 plants ranged from −22‰ to −34‰ (mean −27‰) (Chen et al., [2007]), while δ^13^C values of *P. nil* plants grown in the modified Hoagland solution with 10 mM NaHCO_3_ which δ^13^C was −17.23‰, were very negative, the δ^13^C values of *P. nil* were all lower than −38‰. This observation suggest that inorganic carbon sources for photosynthesis do not come entirely from the atmospheric CO_2_, the carbon source for photosynthesis of *P. nil* also came from the CO_2_ supplied by the transformation from HCO_3_^−^ through CA, and with higher photosynthetic inorganic carbon assimilation efficiency than the other two species, δ^13^C values of *P. nil* were more negative than those of the other two plants, *P. nil* absorbed and assimilated more CO_2_ translated from HCO_3_^−^. But with increasing P deficiency stress and treatment duration, there was no significant change in δ^13^C values, indicated that long-term P deficiency stress had little effect on the way of inorganic carbon utilization.

### *Lonicera pampaninii* Levl

Even though CA activity of *L. pampaninii* was also very high, however, when *L. pampaninii* was under P deficiency stress, especially under 0 mM P concentration, its photosynthetic inorganic carbon assimilation efficiency still decreased compared with those of the control. Therefore, *L. pampaninii* cannot adapt to P deficiency only by CA regulation. The response of Fv/Fm and Φ_PSII_ in *L. pampaninii* to increased P deficiency indicated that *L. pampaninii* exhibited a poor tolerance under 0 mM P concentration as P deficiency stress increased. The quantum efficiencies in the primary reaction center of the open PSII decreased under 0 mM P concentration as P deficiency stress increased. *L. pampaninii* showed no good adaptability to the short-term P deficiency stress, while *L. pampaninii* exhibited a long-term tolerance under 0.125 mM P concentration, its photosynthetic inorganic carbon assimilation ability was inhibited when it was under P deficiency stress especially under 0 mM P concentration.

With the regulation of high CA activity, *L. pampaninii* could assimilate CO_2_ which was transformed from HCO_3_^−^, since the δ^13^C value of CO_2_ which was transformed from HCO_3_^−^ was more negative, δ^13^C in *L. pampaninii* was around −34‰ which was more negative than the average (−27‰) of C3 plants. But less CO_2_ which was transformed from HCO_3_^−^ was assimilated by the photosynthesis process of *L. pampaninii* than *P. nil*, so the δ^13^C of *L. pampaninii* appeared more positive than that of *P. nil*. In addition, with increasing P deficiency stress and treatment duration, there was no significant change in δ^13^C values, indicated that the stomatal conductance of *L. pampaninii* remained constant or the way of inorganic carbon utilization in *L. pampaninii* did not change markedly under P deficiency stress.

### *Parthenocissus tricuspidata*(Sieb.et Zucc.) Planch

The CA activity of *P. tricuspidata* was too low to provide enough carbon through HCO_3_^−^ to CO_2_ transformation. *P. tricuspidata* had a low net CO_2_ assimilation rate and could grow normally with little carbon and P, even under 0 mM P. The response of Fv/Fm and Φ_PSII_ in *P. tricuspidata* to increased P deficiency indicated that *P. tricuspidata* exhibited a long-term tolerance even under 0 mM P concentration. *P. tricuspidata* maintained higher quantum efficiencies in the primary reaction center of the open PSII with longer P deficiency stress durations, especially under 0 mM P. In fact, *P. tricuspidata* grew slowly, its requirement of P was very low, the long-term P deficiency stress had a little effect on the net CO_2_ assimilation rate of *P. tricuspidata*.

The photosynthetic inorganic carbonic ability of *P. tricuspidata* was low, so the carbon available for its photosynthesis was adequate and its photosynthetic inorganic carbon assimilation ability was not significantly inhibited. The δ^13^C of *P. tricuspidata* also appeared more positive than that of *P. nil*. In fact, since its CA activity was very low, little CO_2_ which transformed from HCO_3_^−^ through CA was supplied for the photosynthesis of *P. tricuspidata*, so the carbon source for the photosynthesis of *P. tricuspidata* mainly came from the atmospheric CO_2_ (which δ^13^C was −11‰), then its δ^13^C was around −33‰ and more positive than *P. nil*, but with increasing P deficiency stress and treatment duration, there was no significant change in δ^13^C values, indicated that *P. tricuspidata* kept the same way of inorganic carbon utilization even under P deficiency stress.

## Conclusion

The three climber plants differed in their photosynthetic responses and adaptation mechanisms under P deficiency stress. *P. nil* was tolerant to long-term, severe P deficiency stress. This was shown by its stable PSII and regulation of CA. *L. pampaninii* showed no good adaptability to short-term P deficiency stress, but exhibited a long-term tolerance under 0.125 mM P concentration, the photosynthetic inorganic carbon assimilation ability of *L. pampaninii* was affected by short-term P deficiency stress. *P. tricuspidata* exhibited a good tolerance when exposed to long-term P deficiency stress even under 0 mM P concentration conditions, and a stomatal limitation mechanism might appear to be activated in response to P deficiency stress. Thus, *P. tricuspidata* was tolerant to long-term P deficiency stress. Besides, P deficiency stress had little effect on the way of inorganic carbon utilization of the three climber plants. Different adaptation mechanisms to P deficiency stress should be considered for the selection of species when developing P deficiency-resistant plants.

## Authors’ original submitted files for images

Below are the links to the authors’ original submitted files for images. Authors’ original file for figure 1 Authors’ original file for figure 2 Authors’ original file for figure 3

## Abbreviations

ATP: Adenosine triphosphate
CA: Carbonic anhydrase
ChlF: Chlorophyll fluorescence
DIC: Dissolved inorganic carbon
DW: Dry weight
Φ_PSII_: effective quantum yield of PSII
*L. pampaninii**Lonicera pampaninii*: Levl
Fv/Fm: Maximum quantum yield of PSII
An: Net CO_2_ assimilation rate
Planch (*P. tricuspidata*) *Parthenocissus tricuspidata*: (Sieb.et Zucc*.*)
Choisy (*P. nil*) *Pharbitis nil*: (Linn*.*)
P: Phosphorus
PAR: Photosynthetic active radiation
PSII: Photosystem II
RuBP: Ribulose-1,5-bisphosphate
δ^13^C: Stable carbon isotope ratio
