# Supplementary figures and images for: Effect of phosphorus deficiency on photosynthetic inorganic carbon assimilation of three climber plant species
Source: Bot Stud. 2014 Aug 1;55:60. doi: 10.1186/s40529-014-0060-8 (PMC5430359; doi:10.1186/s40529-014-0060-8)

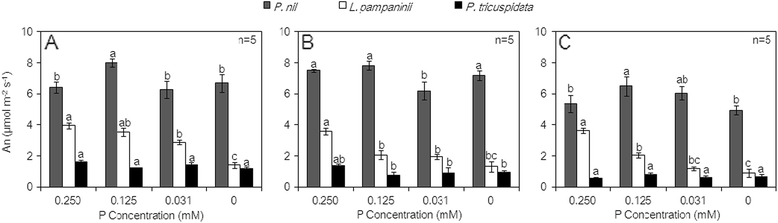

Supplement: Supplementary file 1 — Authors’ original file for figure 1 [file 40529_2014_9060_MOESM1_ESM.gif]

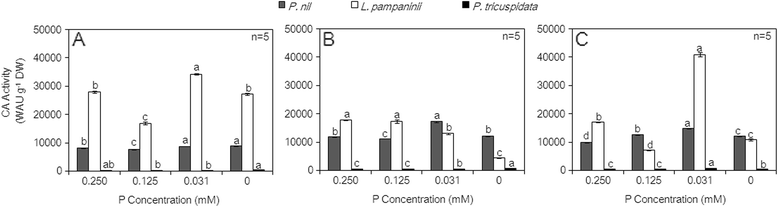

Supplement: Supplementary file 2 — Authors’ original file for figure 2 [file 40529_2014_9060_MOESM2_ESM.gif]

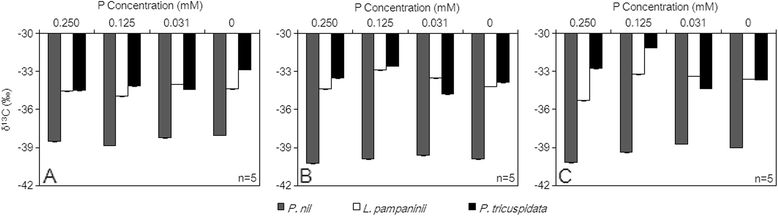

Supplement: Supplementary file 3 — Authors’ original file for figure 3 [file 40529_2014_9060_MOESM3_ESM.gif]
